# Burnout Determinants among Nurses Working in Palliative Care during the Coronavirus Disease 2019 Pandemic

**DOI:** 10.3390/ijerph18073358

**Published:** 2021-03-24

**Authors:** José Vítor Gonçalves, Luísa Castro, Guilhermina Rêgo, Rui Nunes

**Affiliations:** 1 Physical Medicine and Rehabilitation Department, Centro Hospitalar Vila Nova de Gaia/Espinho, 4434-502 Vila Nova de Gaia, Portugal; 2 Faculty of Medicine, University of Porto, 4200-319 Porto, Portugal; guilherminarego@med.up.pt (G.R.); ruinunes@med.up.pt (R.N.); 3 Health Information and Decision Sciences Department—MEDCIDS, Faculty of Medicine, University of Porto, 4200-319 Porto, Portugal; luisacastro@med.up.pt; 4 Center for Health Technology and Services Research (CINTESIS), University of Porto, 4200-319 Porto, Portugal

**Keywords:** burnout, palliative care, nurses, COVID-19, delivery of health care

## Abstract

Nurses working in palliative care are at risk of burnout. The Copenhagen Burnout Inventory was used to determine burnout levels of nurses working in the Portuguese national network of palliative care. We evaluated the contribution of personal, work, and COVID-19 variables in three burnout subclasses: personal, work, and patient-related. A cross-sectional, exploratory, and quantitative design was employed and participants were sampled using convenience and snowball technique. An online survey was conducted and 153 nurses participated in our study. Socio-demographic characterization was conducted, levels of burnout and determinants were explored through multiple linear regression models for its three dimensions. High levels of personal, working, and patient burnout were present in 71 (46%), 68 (44%), and 33 nurses (22%), respectively. Most of them agreed that COVID-19 had an impact on their activities. Significant personal and work related burnout factors found were specialization in palliative care, self-perceived health status, unit type, weekly hours of work, and allocation to COVID-19 units. Gender was found to be a significant factor in patient-related burnout. There is a high level of burnout among nurses working in the Portuguese national network of palliative care. Measures for identification and mitigation of burnout are necessary to protect health care professionals.

## 1. Introduction

According to the World Health Organization (WHO), average life expectancy has been increasing successively and gradually around the world [1]. The improvement in infant mortality indicators and achievements in the treatment of different infectious diseases have allowed developing countries to recover part of the gap in life expectancy in comparison with developed countries [1]. In developed countries, an increase in average life expectancy is associated with a decrease in mortality in the older population [1]. The real impact of the coronavirus disease 2019 (COVID-19) pandemic is not yet known. However, it is responsible for accentuating the discrepancies in access and availability to health care [2] between developed and developing countries. Due to the gradual aging of the worldwide population, different health systems are confronted with both new problems as well as the need to offer new solutions for this growing parcel of the population [3]. According to the WHO, there were 703 million people worldwide aged 65 years or more in 2019, and this number is estimated to double by 2050 [3]. Thus, numerous challenges concerning the needs of the elderly population are threatening different health systems worldwide. Along with the gradual aging of the population, an increasing number of pathologies and clinical situations requiring palliative care (PC) support are also prevalent.

PC is defined by the WHO as care aimed at improving the quality of life of patients and their families facing problems due to incurable and/or serious illness, through the prevention and relief of suffering, by early identification and treatment of several problems: physical, pain, physiological, social, and spiritual [4]. With the development of this kind of care, the myth that PC was only related to terminal oncological situations has started to fade. In fact, several conditions that are not terminal or oncological may receive the support of PC [5].

National Network of Palliative Care (NNPC) in Portugal was created as a solution to the increase in the demand of PC in the country. This network integrates different typologies of healthcare units [6]. Palliative care units (PCUs) are units of inpatient care that may belong to the Portuguese National Health Service (NHS). Despite being private units, they have an agreement with the NHS to integrate with the NNPC. Hospital support palliative care teams (HSPCT) provide support to the hospitals to which they belong, as well as to patients, their families, and/or informal caregivers. Home palliative care teams (HPCT) provide support in the community, to the patients in their homes as well as to their families or informal caregivers [7]. These teams integrate many different palliative care practitioners, such as nurses, doctors, psychologists, social workers, physiotherapists, and occupational therapists [8]. Working teams in which PC nurses are included face a lot of challenges in their demanding clinical activity as, for example, the constant need of scientific updating [9], ethical dilemmas concerning the natural evaluation of the majority of their patients [10] or dealing with expectations, complaints, anxieties and suffering from the terminally ill patients [11,12]. On the other hand, the lack of human resources in the national palliative care naturally leads to increased workload for these health care professionals [8] like nurses. PC services also have higher rates of mortality and that increases the risk of burnout in health care professionals, which is even worse in nurses due to their typical deeper relationship with patients and their families in comparison with other healthcare professionals in PC. As a result of all these challenges, there is a risk of burnout among these nurses in PC [13,14,15].

Freudenberger [16] was one of the first authors to describe the symptoms of exhaustion and burnout, and outline the impact of burnout in health professionals as a series of non-specific symptoms usually in persons working in helping professions. Later, with Maslach and Leiter [17], the concept of burnout evolved as not a crisis with helping or working with people, but as a crisis with the environment at work when coping strategies were no longer effective [17,18]. In this way, burnout can be defined as a “state of physical, emotional, and mental exhaustion that results from long-term involvement in work situations that are emotionally demanding” [19]. Maslach and Jackson [20] also defined burnout syndrome through three dimensions: emotional exhaustion, depersonalization, and a lack of personal accomplishment in a work environment with ineffective coping strategies. Concerning burnout syndrome, it is also important to focus on the consequences of this syndrome for patients. Healthcare professionals with burnout show, for example, a reduction in their performance at work, a greater probability of errors, or lowered job satisfaction [21]. Regarding PC practitioners, nurses and physicians are the most prevalent healthcare workers because of their functions. For this reason, the authors of this national study decided to conduct this study with them; however, this article is only focused on nurses. Some studies have shown that burnout syndrome could be less prevalent among nurses working in PC as compared to nurses working in other fields [22]. However, it is important to be aware of the impact of burnout in nurses by identifying vulnerable professionals and implementing precocious measures to avoid or mitigate burnout. It is also important to remember that it is only possible to have satisfied patients and a high quality care when we have satisfied and committed professionals [23].

Very little is known about the impact of COVID-19 pandemic in burnout in PC nurses. These pandemic months were rough, and difficult times for healthcare professionals worldwide, and a time of significant adversity and challenge for them that is not yet finished. Therefore, the purpose of this study was to evaluate the levels and the determinants of burnout of nurses working in NNPC in Portugal. It is also important to acknowledge that as far the authors know and could confirm, this article is the first worldwide and in Portugal to evaluate the levels and the determinants of burnout among nurses in PC during the COVID-19 pandemic. Hence, this study is a great contribution to determine the impact of COVID-19 pandemic in nurses in PC.

## 2. Materials and Methods

The aim of this study was to evaluate and analyze the burnout levels of nurses working in NNPC in Portugal using the Copenhagen Burnout Inventory (CBI) [24]. In addition, this study also evaluated whether there is any significant contribution of different variables in this population to the three burnout dimensions described previously.

### 2.1. Research Participants

Target population of this study were all the PC nurses working in NNPC in Portugal.

### 2.2. Study Design

In this cross-sectional, exploratory, and quantitative study, nurses working in NNPC in Portugal were sampled by convenience and snowball technique. This study was approved by the Ethics Committee of São João Hospital Center (Ref 195/2020 on 15 June 2020) and followed ethical procedures in accordance with the Declaration of Helsinki. All participants received informed consent online in accordance with the General Data Protection Regulation Guidelines [25]. The author responsible for the validation of the CBI for Portuguese language also gave authorization for the use of his scale in this study. Inclusion criteria of this study were all the nurses working in NNPC in Portugal. A questionnaire was built on Google^®^ Forms (Google^®^, Mountain View, CA, USA) comprising a section with socio-demographic and professional questions, followed by the Portuguese validated version [26] of the CBI [24]. There was not any missing data in this study. Response rate of this study was difficult to estimate as there are not any official document concerning the total number of nurses working in NNPC.

The survey was sent to the institutional e-mails of all the teams that work in NNPC in Portugal [27] and also distributed via social networks (Linkedin^®^ (Microsoft^®^, Mountain View, CA, USA) and Facebook^®^ (Facebook Inc.^®^, Menlo Park, CA, USA) The institutional e-mails of all the teams were available in the Portuguese health ministerium. The questionnaire was available for responses online and data were collected from 20 July 2020 to 1 November 2020. This project was supported by the Order of Portuguese nurses and the Portuguese Association of Palliative Care who distributed this questionnaire. Personal and work-related variables (including the impact of COVID-19) were collected in this survey using a self-administered questionnaire developed by the authors of this study. Personal variables collected were gender, age, marital status, parental responsibility of underage children, weekly physical exercise, and self-perceived health status. Work-related variables collected were academic degree, exclusive dedication in PC, years of activity in PC, unit type as place of work in NNPC, and weekly hours in PC. Regarding the impact of COVID-19 on their work, nurses were asked whether their activity in PC was affected by COVID-19 and if they were shifted from PC activity to COVID-19 units.

### 2.3. Instruments Used

Burnout was measured using the Portuguese validated version of the CBI [26]. This CBI scale has 19 questions that are related to three subscales of burnout: personal, work-related, and client-related (reformulated to patient-related in this study). Questions were answered on a 5-point Likert response scale—each question of the different subscales have 5 different and possible answers scoring 0, 25, 50, 75 or 100 according to the authors’ instructions.

The personal burnout subscale (six questions) evaluates the degree of physical, physiological, and personal sensation of exhaustion, e.g., “How often do you feel tired?”. The work-related burnout subscale (seven questions) evaluates the degree of physical and psychological fatigue and the personal sensation of exhaustion toward work, e.g., “Do you feel burnt out because of your work?”. The patient-related burnout (client/patient) subscale (six questions) assesses the degree of physical and psychological fatigue and personal sensation of exhaustion related to working with patients, e.g., “Do you find it hard to work with patients?”. The total score for each subscale is the calculated mean of the scores of that subscale’s answers, ranging from 0 to 100. If the total score of that subscale is equal to or greater than 50, it is considered a high-level of burnout for that subscale [22,24]. All subscales have high internal consistency with a Cronbach’s alpha (α) in the original version ranging from α = 0.84 to α = 0.87. In the Portuguese version, the α for personal burnout was 0.845, for work-related burnout was 0.866, and for client-related burnout was 0.843 [24,26]. In our sample, α was 0.887, 0.886, and 0.870, respectively.

### 2.4. Statistical Methods

All the data from Google^®^ Forms were exported to a Microsoft Excel 2016 spreadsheet, USA, and data analysis was performed using SPSS^®^ Statistics (version 26.0; SPSS Inc., Chicago, IL, USA) and Jamovi software (The jamovi project (2021). Jamovi^®^ (Version 1.6) (Computer Software) Sydney, Australia). To describe the categorical variables, absolute and relative frequencies, *n* (%), were used. In the case of a normally distributed quantitative variable, the mean and standard deviations were used to describe the variables. If the quantitative variable was non-normally distributed, these variables were described using medians and interquartile intervals (Q1, Q3). To facilitate a comparison with other studies, the mean and standard deviation were also described in these cases. The observation of histograms allowed for verification of normality. For each independent variable (personal, work, and COVID-19 related), a simple linear regression was performed for each outcome of interest: personal burnout, work-related burnout, and patient-related burnout. If the variables were related to the outcomes (*p* ≤ 0.20), these variables were included in the multiple linear regression analyses for each outcome (personal burnout, work-related burnout and patient-related burnout). In the final model, only the significant (*p* ≤ 0.05) independent variables were maintained for each outcome and the results of linear regressions are shown with unstandardized coefficients (B), 95% confidence intervals (95% CI), and *p*-values. Standardized β and semi-partial squared-correlations (sum-of-squares of the effect divided by the total sum-of-square) for the final models are also presented. The final multivariable models were evaluated using F statistics, *p*-values and coefficients of determination (R^2^).

The assumptions of the linear regression models were verified as follows: normality of residuals was assessed by visual analysis of histograms, *t*-tests were employed to test if average residuals were zero, and homoscedasticity was checked using scatter plots of residuals versus the predictive values. In all tests, values of *p* ≤ 0.05 were considered significant.

## 3. Results

During the period of data collection, 153 nurses working in NNPC in Portugal participated in our study. Most of them were women (*n* = 133, 86.9%), and the median (Q1, Q3) age of all nurses was 39 (35, 48) years old. A total of ninety-three nurses (60.8%) had underage children, and 101 nurses (66%) were married or in a civil union. Most of them (*n* = 99, 64.7%) worked entirely in PC with a specialization in PC (*n* = 119, 77.8%). A total of fifty-five nurses (35.9%) worked in HPCT, 48 nurses (31.4%) worked in HSPCT, and 50 nurses (32.7%) worked in PCU. A majority of the nurses (*n* = 135, 88.2%) believed that their clinical activity in PC was affected by COVID-19, and the vast majority were not allocated to other clinical functions in COVID-19 units (*n* = 131, 85.6%). With regard to working hours in PC, 29 nurses (19%) reported working less than 20 weekly hours, 100 nurses (65.4%) worked between 20 to 40 weekly hours, and 24 nurses (15.7%) worked more than 40 weekly hours. Regarding the number of years working of in PC, the median (Q1, Q3) was 5 (3, 9) years. Most of the nurses reported good health status (*n* = 103, 67.3%), but almost half (40%, *n* = 61) did not report any regular physical activity. The local residence of nurses was divided into seven regions determined by the Portuguese Territorial Units for Statistics Level II (NUTS II): North of Portugal (*n* = 53, 34.6%), Center of Portugal (*n* = 38, 24.8%), Lisbon (*n* = 46, 30.1%), Alentejo (*n* = 11, 7.2%), Algarve (*n* = 4, 2.6%), and Autonomous Region of Azores (*n* = 1, 0.7%). No answers were received from the Autonomous Region of Madeira. The full characterization of all participants is summarized in Appendix A, Table A1.

The levels of burnout of the nurses in this study for each subscale were divided between low and high levels of burnout, with a low level of burnout assigned to a final score below or equal to 50 and a high level of burnout assigned to a final score above 50. High levels of personal burnout were found in 71 nurses (46%), high levels of work-related burnout were present in 68 nurses (44%), and high levels of patient-related burnout were present in 33 nurses (22%).

The personal, work, and COVID-19 variables that were eligible for the multiple linear regression models (*p* ≤ 0.20 in the simple regression) were similar for each subscale of burnout (Table 1). For work-related burnout and patient-related burnout, the variables included gender, academic degree, exclusive dedication and specialization in PC, self-perceived health status, unit type of PC, weekly hours of work in PC, and variables related to COVID-19. For personal burnout, the variables were the same, except for gender (Table 1).

In the final multiple model, for personal burnout and work-related burnout, the significant variables were specialization in PC, self-perceived health status, unit type of PC, and allocation to COVID-19 units. The final models explained approximately 30.3% and 31.3% of the total data variance of personal and work-related burnout, respectively. Self-perceived health status represented the most relevant variable in terms of variance explained for personal burnout while for work-related burnout the most contributing variable was the PC type of unit (Table 2).

For patient-related burnout, the analyzed variables in the final model were the same, with an addition of gender, explaining approximately 32.6% of the total variability in patient-related burnout (Table 3), where gender and self-perceived health status represented the most relevant variables in the model in terms of variance explained (Table 2). No problems of multicolinearity were present, as the final model for personal burnout and work-related burnout presented variance inflation factors (VIF) between 1.03 and 1.06, and between 1.02 and 1.06 for patient-related burnout.

The personal burnout levels of nurses who perceived their health status as very good or good were, respectively, 17.11 and 9.27 points lower on average in comparison with nurses who perceived their health as not good or bad, respectively. Nurses with specialization in PC had lower levels of personal burnout, with a reduction on average of 7.56 and 8.88 points for post-graduation and master or PhD degree, respectively, as compared to nurses with no specialization. Nurses who worked in HSPCT and PCU (private institutions) had, on average, 7.55 and 14.74 higher levels of personal burnout as compared to nurses working in HPCT. Nurses who were shifted from PC to COVID-19 units had, on average, 7.92 more points in personal burnout as compared to nurses who were not shifted to COVID-19 units.

Work-related burnout levels of nurses who perceived their health status as very good or good were 14.57 and 9.10 points lower on average, respectively, as compared to nurses who self-perceived their health as not good or bad. Nurses who had specialization in PC showed lower levels of work-related burnout with a reduction on average of 11.24 and 15.20 points for post-graduation and master or PhD degree, respectively, as compared to nurses with no specialization. Nurses who worked in HSPCT, PCU (private) and PCU (NHS) had, on average, 11.01, 16.50, and 10.31, respectively, higher levels of work-related burnout as compared to nurses working in HPCT. Nurses who were shifted from PC to COVID-19 units had, on average, 9.80 more points of work-related burnout in comparison with nurses who were not allocated to COVID-19 units.

Patient-related burnout levels were 15.97 points lower, on average, among women as compared to men. Patient-related burnout levels of nurses who perceived their health status as very good or good were 14.94 lower, on average, in comparison with nurses who perceived their health as not good or bad. Nurses who specialized in PC had lower levels of patient-related burnout, with an average reduction of 10.79 points for master’s or PhD degree as compared to nurses with no specialization. Nurses working at HSPCT and PCU (private and NHS institutions) had, on average, 7.73, 12.84 (private) and 9.29 (NHS) higher levels of patient-related burnout as compared to nurses working in HPCT. Nurses who were shifted from PC to COVID-19 units had, on average, 14.87 more points in patient-related burnout than nurses who were not allocated to COVID-19 units. These results are summarized in Table 3.

## 4. Discussion

In our study, there was a significant difference between the prevalence of high levels in personal burnout, work-related burnout and patient-related burnout. The prevalence of high levels personal burnout and work-related burnout were 46% and 44%, respectively. On the other hand, the prevalence of high levels of patient-related burnout was 22%. Our study showed higher levels of burnout as compared to other prior studies [28]. It is important to remember that this study was conducted during the COVID-19 pandemic, which clearly had an impact worldwide and on healthcare workers like nurses [29]. This situation could have influenced the results of this study, causing higher levels of burnout being reported. The results of this study also support the conclusions in the literature about the impact of COVID-19 on non COVID-19 diseases such as PC assistance, as most nurses (*n* = 135, 88.2%) agreed to the fact that COVID-19 had an impact on their PC activity [30]. Some nurses in this study were also shifted to COVID-19 units, which could have also had an impact on the results of our investigation (*n* = 22, 14.4%) [31]. In fact, the levels of burnout in this study were similar to other studies that tried to assess the impact of COVID-19 on burnout among healthcare workers worldwide [32]. Our study suggests that there is a large size association between allocation to COVID-19 units and the burnout levels (Table 2) but this overall association can be related to different variables that were not evaluated in our study like depression [33], resilience [33], stress [32], existence of previous psychological problems [34] or had a traumatic event in relation with COVID-19 like previous infection [34]. World Health Organization defines health as “a state of complete physical, mental and social well-being and not merely the absence of disease or infirmity.” [35]. Being moved to a COVID unit could have had an impact on one or more of these elements and led to poor health status as described before. However, the possibility of an interaction between the two independent variables “being moved to the COVID unit” and “self-assessed health” was analyzed and it was non-significant in the final model, for each one of the three burnout dimensions. There were not found in the literature so far any more studies like ours that tried to evaluate the burnout level in nurses in PC after the outbreak of COVID-19 worldwide. Therefore, long as the COVID-19 pandemic endures, it will continue to be a source of stress among nurses and all other healthcare professionals. Hence, it is mandatory to offer healthcare professionals’ answers and measures that promote their wellbeing and mental health in the following months [36]. The high levels of burnout in this study were according to the results of other studies in the literature that also presented high levels of burnout [28,32,37]. However, it is important to highlight that there are few studies in the literature related to burnout among nurses in PC and most of them used the Maslach Burnout Inventory, making it more difficult to compare the results.

The most recent national report of NNPC also supports our conclusions about the gender, median age of the nurses, number of years of working in PC, and weekly hours of work in NNPC [8]. The results of this report are similar to the previously identified variables utilized in our study. For the three burnout dimensions, the personal, work, and COVID-19 variables that were included in the final model were almost the same, except for gender, which was only present in patient-related burnout. Patient-related burnout refers to the individual’s exhaustion in relation to their work with clients. In our study, as previously mentioned, the clients were patients and the concept of patient-related burnout was related to the degree of exhaustion that the individual relates to their work with patients. This depersonalization leads to detachment from patients, co-workers as well as with the organization itself [38]. It is important to remember that individuals could experience other sources of fatigue and exhaustion, in addition to working with patients. In our final model of this study, women were associated with lower levels of patient-related burnout. In theory, female nurses could show higher levels of burnout and patient-related burnout due to a double burden that still exists in society. Women balance their work as nurses with their unpaid domestic labor at home, i.e., house management or dealing with their children. Some studies showed higher levels of burnout among female workers [39,40]; however, in our study, patient-related burnout levels were lower in female nurses. In fact, some studies suggest that women have lower levels of patient-related burnout than men [41]. Some of the explanations for this phenomenon are related to stereotypical gender roles in society. In some societies, the role of nurses is still associated with women as an act of caring in complementarity with the role of mother and wife. On the other hand, men are expected to be distant with regard to their emotions and uninvolved in workplace relationships [42,43]. The other variables suggested as predictors for personal burnout, work-related burnout, and patient-related burnout in our results were specialization in PC, self-perceived health status, unit type of PC, and nurses’ allocation to COVID-19 units. According to our final model, specialization in PC was significantly associated with less burnout in all subscales (although regarding patient-related burnout, the postgraduate category was not statistically significant). These results are also supported by prior literature, and the specialization in PC is associated with acquiring skills and knowledge on managing situations that could lead to burnout [44]. In terms of self-perceived health status, the notion of a very good or good health status was related to less burnout in personal burnout and work-related burnout. In relation to patient-related burnout, only the self-perceived status of very good health was significantly associated with lower levels of burnout. These results were in line with prior literature as well as with our expectations, [45] as nurses who think that they are in a better health situation are more likely to be able to deal with situations that could increase the levels of burnout. According to the unit type of PC, the levels of burnout were different—average levels of burnout of nurses working in the PCU that were in agreement with the NHS were higher. In fact, the levels of personal burnout, work-related burnout, and patient-related burnout were 14.74, 16.50, and 12.84, respectively, higher on average, than that of HPCT in these units. The levels of personal burnout, work-related burnout, and patient-related burnout in PCUs that belong to the NHSS were 5.68, 10.31, and 9.29, respectively, which were higher than those of HPCT. These differences could be related to the organization of NNPC in NHS [6]. PCUs that are in agreement with the NHS are completely private institutions. Some of them are not traditional hospitals and each one has their own working environment and organization that is very different from hospitals. On the other hand, PCUs that belong to the NHS are all traditional hospitals and their burnout levels results are different. The differences in the work environment and the possibility of having other support from the classical structure of a hospital could be one of the reasons for the differences in the PCUs [46]. Higher levels of personal burnout, work-related burnout, and client-related burnout of HSPCT in comparison with HPCT could also be related to the fact that despite being within the hospital with theoretically better support than in the community, these teams must support every patient in every department of the hospital. The constant need and multiple calls for each patient could contribute to higher levels of burnout [47]. 

The originality of this article during the COVID-19 pandemic could be an opportunity to have a real impact on PC nurses’ activity and their patients as it offers the first perspective of the new reality of these healthcare professionals during this COVID-19 pandemic. The majority of nurses in this study think that COVID-19 had an impact on their PC activity in non-COVID-19 patients. Besides, and concerning the results of this study, measures should be taken to measure and avoid mainly personal and work-related burnout. If the PC nurses have fewer levels of burnout and less sensation of the impact of COVID-19 in their clinical activity, their patients will also benefit from better care. Despite the interesting conclusions of this study, some limitations should be considered. The first is related to the impact of COVID-19 pandemic on burnout levels. The entire period of data collection was through the COVID-19 pandemic crisis and that definitely had an impact on burnout levels. Second, the other limitation is hypothetical in nature, as nurses who were less familiar or adept at using the Internet could have not been represented in the sample. Third, the huge difference in the gender representation in this sample could have an influence in gender related variables results. Forth, this study was a cross sectional one with no follow up of the nurses over time and during the COVID-19 pandemic and there is the need for future longitudinal studies related to the impact of COVID-19 in nurses’ burnout. Fifth, other variables related to COVID-19 could influence these results like the infection of the participants of their relatives during the time of this study. Finally, despite being completely anonymous, participants could have a tendency to rate themselves better in terms of levels of burnout and dealing with patients.

## 5. Conclusions

The prevalence of high levels of personal burnout, work-related burnout, and patient-related burnout were 46%, 44%, and 22%, respectively. We have found high and higher levels of burnout in our study in comparison with the literature. The overall difference among the subscales was in line with prior literature. The variables that contributed to all the burnout dimensions in the final models were the same in personal burnout and work-related burnout and gender contributed only to patient-related burnout. In the future, it would be interesting to replicate the same study to verify whether the beginning of vaccination to COVID-19 had a positive effect on overall levels of burnout. This study could also be replicated with other healthcare workers, such as physicians of NNPC. The authors of this study are already working on this. Concerning these high levels of burnout, measures should be implemented in the NPCC to identify nurses at risk of burnout and implement measures to mitigate and avoid burnout. This study also suggests the impact of COVID-19 on PC activity.

## Figures and Tables

**Table 1:** Unstandardized regression coefficients of univariable models for the subscales of burnout according to CBI as outcomes and personal, work and COVID-19 variables as predictors.

Variables	Personal Burnout	Work-Related Burnout	Patient-Related Burnout
	B [95% CI]	B [95% CI]	B [95% CI]
Gender			
Male	Ref	Ref	Ref
Female	1.04 [−7.29; 9.37]	−6.53 [−15.2; 2.13] ^+^	−18.3 [−27.7; −8.81] ***
Age (years)	0.03 [−0.28; 0.35]	−0.06 [−0.39; 0.27]	−0.04 [−0.42; 0.33]
Marital status			
Married/Civil union	Ref	Ref	Ref
Divorced/Separated	−0.57 [−8.01; 6.88]	−1.62 [−9.4; 6.17]	−2.49 [−11.30; 6.31]
Single	−0.62 [−8.53; 7.30]	0.91 [−7.37; 9.19]	4.57 [−4.78; 13.93]
Underage children			
No	Ref	Ref	Ref
Yes	−1.37 [−7.12; 4.37]	−3.07 [−9.08; 2.93]	−4.53 [−11.3; 2.27]
Academic degree			
Bachelor	Ref	Ref	Ref
Master	−1.59 [−7.17; 3.99]	−4.54 [−10.4; 1.31] ^+^	−2.11 [−8.78; 4.57]
PhD	−28.27 [−52.80; −3.74] *	−22.65 [−48.4; 3.06] ^+^	−25.66 [−55.01; 3.69] ^+^
Weekly physical exercise			
No regular practice	Ref	Ref	Ref
Less than 75 min	−1.55 [−7.88; 4.78]	−1.39 [−7.99; 5.20]	0.57 [−6.96; 8.10]
75 min or more	0.13 [−7.47; 7.73]	3.81 [−4.11; 11.73]	1.78 [−7.25; 10.82]
Exclusive dedication to PC			
No	Ref	Ref	Ref
Yes	−7.17 [−12.9; −1.41] *	−8.52 [−14.5; −2.52] **	−8.90 [−15.7; −2.07] *
Self-perceived health status			
Not good nor bad	Ref	Ref	Ref
Bad or very bad	17.92 [3.67; 32.16] *	13.10 [−2.39; 28.58] ^+^	22.08 [4.46; 39.71] *
Good	−9.94 [−16.55; −3.33] **	−9.32 [−16.50; −2.13] *	−5.53 [−13.71; 2.64] ^+^
Very good	−18.79 [−29.10; −8.48] ***	−15.39 [−26.60; −4.19] **	−15.52 [−28.27; −2.76] *
Years of activity in PC	0.16 [−0.48; 0.79]	−0.08 [−0.75; 0.59]	0.02 [−0.75; 0.79]
Specialization in PC			
No	Ref	Ref	Ref
Bachelor’s degree or post-graduation	−7.66 [−14.9; −0.37] *	−11.1 [−18.6; −3.59] **	−7.10 [−15.8; 1.59] ^+^
Master’s or PhD degree	−6.58 [−14.1; 0.91] ^+^	−11.5 [−19.2; −3.83] **	−7.91 [−16.8; 1.02] ^+^
PC type of unit			
HPCT	Ref	Ref	Ref
HSPCT	4.79 [−1.79; 11.4] ^+^	6.60 [−0.27; 13.5] ^+^	5.27 [−2.63; 13.2] ^+^
PCU (Private)	16.76 [8.21; 25.3] ***	17.65 [8.73; 26.6] ***	16.82 [6.55; 27.1] **
PCU (NHS)	3.28 [−4.37; 10.9]	8.26 [0.28; 16.2] *	9.35 [0.16; 18.5] *
Weekly hours of activity			
Less than 20	Ref	Ref	Ref
Between 20 and 40	−6.84 [−14.04; −0.36] ^+^	−7.17 [−14.69; 0.36] ^+^	−7.76 [−16.32; 0.80] ^+^
More than 40	1.00 [−8.42; 10.42]	1.71 [−8.14; 11.56]	1.98 [−9.22; 13.18]
PC affected by COVID−19			
No	Ref	Ref	Ref
Yes	11.1 [2.56; 19.6] *	9.87 [0.88; 18.9] *	6.85 [−3.45; 17.2] ^+^
Allocated to COVID−19 units			
No	Ref	Ref	Ref
Yes	13.2 [5.48; 20.9] ***	13.7 [5.66; 21.8] ***	19.8 [10.8; 28.8] ***

Legend: PC—Palliative Care; HPCT—Home Palliative Care Teams; HSPCT—Hospital Support Palliative Care Teams; PCU—Palliative Care Unities; NHS—National Health Service; Ref—Reference category; B—unstandardized coefficient; CI—confidence interval. Note: ^+^ *p* ≤ 0.20; * *p* ≤ 0.05; ** *p* ≤ 0.01; *** *p* ≤ 0.001.

**Table 2:** Standardized regression coefficients and semi-partial squared correlations in multivariable models for the subscales of burnout according to CBI as outcomes and personal, work and COVID-19 variables as predictors.

Variables	Personal Burnout		Work-Related Burnout		Patient-Related Burnout	
	Standardized β [95% CI]	η^2^	Standardized β [95% CI]	η^2^	Standardized β [95% CI]	η^2^
Gender						
Male	-		-		Ref	0.0645
Female	-		-		−0.767 ***	
Self-perceived health status						
Not good or bad	Ref	0.1198	Ref	0.0716	Ref	0.0613
Very good	−0.976 ***		−0.794 **		−0.717 *	
Good	−0.529 **		−0.496 **		−0.258	
Very bad or bad	0.780		0.381		0.695	
Specialization in PC						
None	Ref	0.0347	Ref	0.0850	Ref	0.0315
Post-graduation	−0.432 *		−0.615 ***		−0.307	
Master’s or PhD degree	−0.507 *		−0.828 ***		−0.518 *	
PC type of unit						
HPCT	Ref	0.0706	Ref	0.0989	Ref	0.0475
HSPCT	0.431 *		0.600 **		0.371 *	
PCU (Private)	0.841 ***		0.899 ***		0.617 **	
PCU (NHS)	0.324		0.562 **		0.446 *	
Allocated to COVID-19 units						
No	Ref	0.0228	Ref	0.0318	Ref	0.0566
Yes	0.452 *		0.534 *		0.714 ***	
R^2^	0.303		0.313		0.326	
F	6.90 ***		7.23 ***		6.88 ***	

Legend: PC—Palliative Care; HPCT—Home Palliative Care Teams; HSPCT—Hospital Support Palliative Care Teams; PCU—Palliative Care Unities; NHS—National Health Service; Ref—Reference category; β—standardized coefficient; CI—confidence interval; R^2^—Determination Coefficient; F—F statistics. Note: * *p* ≤ 0.05; ** *p* ≤ 0.01; *** *p* ≤ 0.001.

**Table 3:** Unstandardized regression coefficients of multivariable models for the subscales of burnout according to CBI as outcomes and personal, work and COVID-19 variables as predictors.

Variables	Personal Burnout B [95% CI]	Work-Related Burnout B [95% CI]	Patient-Related Burnout B [95% CI]
Gender			
Male	-	-	Ref
Female	-	-	−15.97 [−24.53; 7.40] ***
Self-perceived health status			
Not good or bad	Ref	Ref	Ref
Very good	−17.11 [−27.22; −7.00] ***	−14.57 [−25.08; −4.07] **	−14.94 [−26.78; −3.09] *
Good	−9.27 [−15.53; −3.01] **	−9.10 [−15.60; −2.59] **	−5.36 [−12.70; 1.98]
Very bad or bad	13.68 [−0.14; 27.49]	7.00 [−7.36; 21.36]	14.46 [−1.74; 30.66]
Specialization in PC			
None	Ref	Ref	Ref
Post-graduation	−7.56 [−14.04; −1.09] *	−11.24 [−18.00; −4.54] ***	−6.38 [−13.97; 1.20]
Master’s or PhD degree	−8.88 [−15.93; −1.83] *	−15.20 [−22.53; −7.87] ***	−10.79 [−19.07; −2.51] *
PC type of unit			
HPCT	Ref	Ref	Ref
HSPCT	7.55 [1.03; 14.07] *	11.01 [4.24; 17.79] **	7.73 [−0.09; 15.38] *
PCU (Private)	14.74 [6.77; 22.72] ***	16.50 [8.21; 24.78] ***	12.84 [3.49; 22.19] **
PCU (NHS)	5.68 [−1.29; 12.65]	10.31 [3.07; 17.55] **	9.29 [1.00; 17.59] *
Allocated to COVID-19 units			
No	Ref	Ref	Ref
Yes	7.92 [0.68; 15.17] *	9.80 [2.27; 17.33] *	14.87 [6.36; 23.38] ***
R^2^	0.303	0.313	0.326
F	6.90 ***	7.23 ***	6.88 ***

Legend: PC—Palliative Care; HPCT—Home Palliative Care Teams; HSPCT—Hospital Support Palliative Care Teams; PCU—Palliative Care Unities; NHS—National Health Service; Ref—Reference category; B—unstandardized coefficient; CI—confidence interval; R^2^—Determination Coefficient; F—F statistics. Note: * *p* ≤ 0.05; ** *p* ≤ 0.01; *** *p* ≤ 0.001.

## Data Availability

The data presented in this study are available on request from the corresponding author. The data are not publicly available due to privacy or ethical restrictions.

## Appendix A

**Table A1:** Sample characterization (*n* = 153).

Characteristics	*n*	%
Gender		
Male	20	13.1
Female	133	86.9
Marital Status		
Married/Civil union	101	66
Divorced/Separated	28	18.3
Single	24	15.7
Underage children		
Yes	93	60.8
No	60	39.2
Nationality		
Portuguese	152	99.3
Non-Portuguese	1	0.7
Exclusive dedication to PC		
Yes	99	64.7
No	54	35.3
Specialization in PC		
No	34	22.2
Bachelor’s degree or post-graduation	64	41.8
Master’s or PhD degree	55	35.9
PC type of unit		
HPCT	55	35.9
HSPCT	48	31.4
PCU (Private)	21	13.7
PCU (NHS)	29	19
PC affected by COVID-19		
Yes	135	88.2
No	18	11.8
Allocated to COVID-19 units		
Yes	22	14.4
No	131	85.6
Weekly hours of activity		
Less than 20	29	19
Between 20 and 40	100	65.4
More than 40	24	15.7
Self-perceived health status		
Very good	14	9.2
Good	103	67.3
Not good or bad	30	19.6
Bad or very bad	6	4

Legend: PC—Palliative Care; HPCT—Home Palliative Care Teams; HSPCT—Hospital Support Palliative Care Teams; PCU—Palliative Care Unities; NHS—National Health Service.
